# Texture analysis of T2-weighted cardiovascular magnetic resonance imaging to discriminate between cardiac amyloidosis and hypertrophic cardiomyopathy

**DOI:** 10.1186/s12872-022-02671-0

**Published:** 2022-05-21

**Authors:** Shan Huang, Ke Shi, Yi Zhang, Wei-Feng Yan, Ying-Kun Guo, Yuan Li, Zhi-Gang Yang

**Affiliations:** 1 Department of Radiology, West China Hospital, Sichuan University, No. 37 Guo Xue Xiang, Chengdu, 610041 Sichuan China; 2 Department of Radiology, Key Laboratory of Birth Defects and Related Diseases of Women and Children of Ministry of Education, West China Second University Hospital, Sichuan University, Chengdu, China

**Keywords:** Texture analysis, Cardiac amyloidosis, Hypertrophic cardiomyopathy, Left ventricular hypertrophy

## Abstract

**Background:**

To elucidate the value of texture analysis (TA) in detecting and differentiating myocardial tissue alterations on T2-weighted CMR (cardiovascular magnetic resonance imaging) in patients with cardiac amyloidosis (CA) and hypertrophic cardiomyopathy (HCM).

**Methods:**

In this retrospective study, 100 CA (58.5 ± 10.7 years; 41 (41%) females) and 217 HCM (50.7 ± 14.8 years, 101 (46.5%) females) patients who underwent CMR scans were included. Regions of interest for TA were delineated by two radiologists independently on T2-weighted imaging (T2WI). Stepwise dimension reduction and texture feature selection based on reproducibility, machine learning algorithms, and correlation analyses were performed to select features. Both the CA and HCM groups were randomly divided into a training dataset and a testing dataset (7:3). After the TA model was established in the training set, the diagnostic performance of the model was validated in the testing set and further validated in a subgroup of patients with similar hypertrophy.

**Results:**

The 7 independent texture features provided, in combination, a diagnostic accuracy of 86.0% (AUC = 0.915; 95% CI 0.879–0.951) in the training dataset and 79.2% (AUC = 0.842; 95% CI 0.759–0.924) in the testing dataset. The differential diagnostic accuracy in the similar hypertrophy subgroup was 82.2% (AUC = 0.864, 95% CI 0.805–0.922). The significance of the difference between the AUCs of the TA model and late gadolinium enhancement (LGE) was verified by Delong’s test (*p* = 0.898). All seven texture features showed significant differences between CA and HCM (all *p* < 0.001).

**Conclusions:**

Our study demonstrated that texture analysis based on T2-weighted images could feasibly differentiate CA from HCM, even in patients with similar hypertrophy. The selected final texture features could achieve a comparable diagnostic capacity to the quantification of LGE.

*Trial registration *Since this study is a retrospective observational study and no intervention had been involved, trial registration is waived.

**Supplementary Information:**

The online version contains supplementary material available at 10.1186/s12872-022-02671-0.

## Background

Cardiac amyloidosis (CA) is a progressive and infiltrative cardiomyopathy that frequently leads to heart failure and cardiac death [1]. At present, late gadolinium enhancement (LGE), T1 mapping and T2 mapping on cardiovascular magnetic resonance imaging (CMR) have remarkable performance in diagnosing CA and they could also provide prognostic value for these patients [2]. However, LGE and postcontrast T1 mapping techniques require the administration of gadolinium contrast agent. And the T1 and T2 mapping sequences are not routinely acquired in many institutions.

With the development of “Radiomics”, mining the existing basic imaging data and obtaining useful novel parameters from the radiological images with the use of computer algorithms has become an area of research interest. Texture analysis (TA) quantifies the texture of an image based on spatial distributions of pixel signal intensity and relationships of values between neighboring pixels [3]. TA has the capability to overcome the limitations of traditional subjective visual interpretation of images and recognize lesions that are imperceptible to the human eye. In oncology, TA of CT images has been shown to be able to differentiate different histological characteristics and specific gene mutations in several types of tumors [4, 5]. CMR-based TA has also been reported to have great performance for a few clinical applications. A previous study found that TA of T2 mapping presented high sensitivity and specificity for the diagnosis of acute infarct-like myocarditis [6].

The T2-weighted imaging (T2WI) is widely used to identify pathological lesions in many organs, such as the brain, liver and muscles. Abnormal T2 signal intensity is correlated with edema, cellular proliferation, and vessel densities [7, 8]. In the heart, T2-weighted sequences are often used to identify myocardial edema [9, 10]. However, due to the low contrast between normal and edematous myocardium, it is often a challenge to determine the presence and extent of the lesion [11].

In this preliminary study, we sought to use texture analysis to find novel and useful parameters from the routinely acquired T2WI images, which could discriminate the myocardial tissue of CA from hypertrophic cardiomyopathy (HCM), a subtype of left ventricular hypertrophy (LVH) of different etiology.

## Methods

### Study population

We retrospectively included 317 subjects (CA: n = 100, HCM: n = 217) who underwent CMR scans between January 2016 and June 2020. For patients with CA, most of them were initially diagnosed with systemic disease (such as plasma cell dyscrasia or multiple myeloma) or amyloidosis in other extra-cardiac organs (such as kidney, rectum, subcutaneous fat tissue, etc.). They were referred to CMR to see if the heart was involved. CA was diagnosed on the basis of positive myocardial biopsy or extracardiac biopsy in conjunction with a mean LV wall thickness (septum and posterior wall) ≥ 12 mm on CMR [12]. For CA patients with a subendocardial LGE pattern, results of coronary angiography or computed tomographic coronary angiography, clinical history, and electrocardiography were referred to rule out the possibility of myocardial infarction.

For patients with HCM, they were referred to CMR because of abnormal electrocardiography or left ventricular hypertrophy by echocardiography, as CMR could provide more accurate measurement of morphological alterations and evaluate the extent of myocardial fibrosis. The diagnosis of HCM was based on the presence of unexplained LV wall thickness ≥ 15 mm or ≥ 13 mm with a family history of HCM or apical hypertrophy in the absence of other conditions capable of producing a similar degree of hypertrophy [13]. HCM patients with previous septal ablation or myectomy were excluded.

The exclusion criteria for all subjects were valvular heart disease (greater than mild stenosis or greater than moderate regurgitation), significant coronary artery disease, and other confirmed systemic diseases. Images of poor quality were excluded. Hypertensive patients with concentric hypertrophy were also excluded.

### Cardiovascular magnetic resonance

CMR imaging was performed in accordance to a standard protocol, as previously published by our group [14]. All CMR images were performed using a 3.0 T scanner (MAGNETOM Skyra/Tim Trio; Siemens Healthcare, Erlangen, Germany) with a 30-channel phased-array receiver coil. Cine images were obtained using a balanced steady-state free-precession sequence in consecutive short-axis views covering the entire LV (from the level of the mitral valve annulus to the LV apex) with the following parameters: ﻿temporal resolution, 39.34/42 ms; repetition time (TR), 2.81/3.4 ms; echo time (TE) 1.22/1.3 ms; flip angle, 38°/50°; field of view (FOV), 284 mm × 399 mm; matrix size, 139 × 208; and slice thickness, 8 mm. T2-weighted short inversion time inversion recovery (T2-STIR) imaging was performed with the following parameters: TR, 800 ms/1400 ms; TE, 71 ms/68 ms; flip angle, 180°; FOV, 243 mm × 300 mm; matrix size, 256 × 166; and slice thickness, 8 mm. Late gadolinium enhancement (LGE) imaging was performed at an average of 10–15 min after contrast injection by using a segmented-turbo-FLASH–phase-sensitive inversion recovery (PSIR) sequence (TR/TE = 750.00/1.18 ms; flip angle, 40°; slice thickness, 8 mm; FOV, 400 mm × 270 mm; and matrix size, 184 × 256).

### Imaging analysis

Conventional CMR parameters including LV end-diastolic volume (EDV), LV end-systolic volume (ESV), LV ejection fraction (LVEF), LV mass, maximum LV wall thickness (MWT) and LGE extent were calculated using commercially available software (CVI^42^; Circle Cardiovascular Imaging, Inc., Calgary, Canada). The extent of LGE was quantified by using the full width half maximum technique [15, 16].

**Fig. 1:**
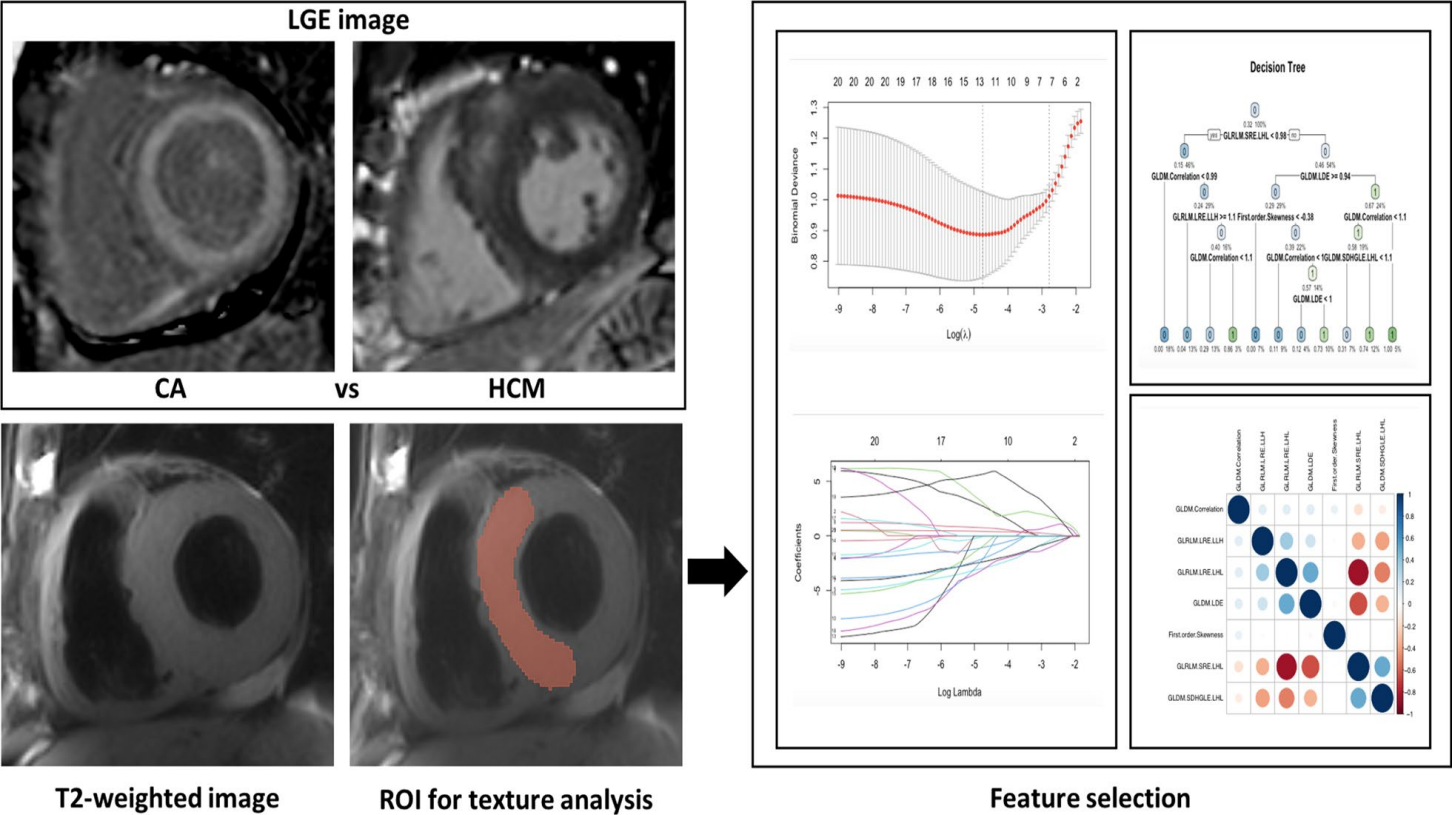
Framework of this study. The extent of LGE in CA and HCM were quantified using the full width half maximum technique. T2WI images were used for the texture analysis. Feature extraction was performed on the ROIs on the basal septum of the left ventricle. Stepwise methods were performed to selecting the optimal features. LGE: late gadolinium enhancement; CA: cardiac amyloidosis; HCM: hypertrophic cardiomyopathy; ROI: region of interest

Texture analysis was performed on T2-weighted CMR scans using 3D Slicer based on the Pyradiomics library [17]. The regions of interest (ROIs) were manually delineated in the basal septum of the left ventricle by a radiologist with 4 years of experience in cardiovascular imaging who was blinded to the patients’ information. ROI delineation was repeated twice in a subset of 30 randomly selected patients by the same radiologist for intraobserver analysis and by another radiologist with 15 years of experience for interobserver analysis. Figure 1 presents the framework of this study.

### Feature extraction and selection

A total of 837 features were extracted from T2-weighted images during the process of image filtering and feature extraction. Stepwise feature selection and dimension reduction were performed due to the high number of texture features. First, the intraclass correlation coefficient (ICC) was calculated to assess the intra- and interobserver reproducibility of the selected features. Features with ICCs < 0.75 were excluded, ICCs ranging from 0.75 to 1 considered “excellent” [18]. The Boruta algorithm [19], corrplot by carret [20] and the least absolute shrinkage and selection operator (LASSO) with tenfold cross-validation [21] were performed in a stepwise manner for dimension reduction. Furthermore, both the CA and HCM groups were randomly divided into a training dataset and a testing dataset (7:3). To select variables that allow for the discrimination of myocardial tissue alterations in HCM and CA patients, the classification tree model [22], a commonly used machine learning algorithm, was employed to calculate discriminative performance in the training cohort and validated in the testing cohort.

After the radiomics signature was established, the diagnostic efficiency and accuracy of the model was validated in patients with similar hypertrophy matched by LV mass index as well as age, sex and maximum wall thickness (MWT).

### Statistics analysis

Statistical analysis during the construction of the radiomics signature was performed in R (version 4.0.1; R Foundation for Statistical Computing, Vienna, Austria) [23] with RStudio (version 1.3.959; RStudio, Boston, Mass) [24]. The R packages used for statistical analyses are described in the Additional file 1. Other statistical analyses were conducted with SPSS (Version 19; IBM, Armonk, NY). The normality of the data distribution was determined using the Kolmogorov–Smirnov test. Continuous data are expressed as the means ± SDs or medians with interquartile ranges. The t-test or the Mann–Whitney U-test was conducted, as appropriate. The diagnostic accuracy of the optimal radiomic parameters was evaluated by the area under the curve (AUC) from receiver operating characteristic (ROC) analyses. The diagnostic sensitivity, specificity, positive predictive value (PPV), negative predictive value (NPV) and accuracy were also calculated. ROC curves were compared using DeLong’s test. P < 0.05 was considered statistically significant.

## Results

### Clinical characteristics of the included patients

The clinical and CMR characteristics of the 317 included patients (CA: n = 100, 41% female, 58.5 ± 10.7 years; HCM: n = 217, 46.5% female, 50.7 ± 14.8 years) are presented in Table 1. The CA group was included based on myocardial biopsy (n = 15, 15%) and extracardiac biopsy: bone morrow (n = 59, 59%), fat (n = 16, 16%), kidney (n = 5, 5%), rectum (n = 4, 4%), and tongue (n = 1, 1%). In addition, 93% of the CA patients were the light-chain amyloidosis type.

**Table 1:** Demographic and clinical characteristics of the included subjects

	CA (n = 100)	HCM (n = 217)	P values
Age, years	58.5 ± 10.7	50.7 ± 14.8	< 0.001
Gender, females, n(%)	41 (41%)	101 (46.5%)	0.318
BMI, kg/m^2^	22.0 ± 3.3	24.1 ± 3.5	< 0.001
NYHA III-IV, n(%)	64 (64%)	23 (10.6%)	< 0.001
Hypertension, n(%)	16 (16%)	65 (30%)	0.001
Diabetes, n(%)	9 (9%)	18 (8.3%)	0.263
Dyslipidemia, n(%)	7 (7%)	12 (5.5%)	0.823
Sub-types of HCM			
Asymmetrical/LOVOT	37	168/124	
Concentric	63	36	
Mid-ventricular	–	2	
Apical	–	11	
AL, n(%)	93 (93%)	/	
NT-proBNP, pg/ml	8.5 ± 1.1	7.0 ± 1.0	< 0.001
Troponin T, pg/ml	4.6 ± 0.7	3.0 ± 0.9	< 0.001
Medications			
Diuretics	68 (68%)	26 (12%)	
β-blockers	26 (26%)	78 (35.9%)	
ACEI/ARB	12 (12%)	31 (14.2%)	
Ca^2+^ blockers	5 (5%)	33 (15.2%)	
Statin	8 (8%)	22 (10.1%)	
Cardiovascular magnetic resonance			
LV-ESV index, ml	26.6 ± 9.5	23.9 ± 9.2	0.029
LV-EDV index, ml	49.6 ± 11.4	59.8 ± 13.8	< 0.001
LVEF, %	46.6 ± 13.0	61.0 ± 7.9	< 0.001
LV mass index, g/m^2^	43.3 ± 13.3	45.6 ± 17.5	0.25
MWT	15.4 (14.5, 17.6)	18.5 (16.2, 21.1)	< 0.001
Presence of main LGE pattern			
Patchy	17 (24.3%)	163 (75%)	
Transmural	25 (35.7%)	54 (25%)	
Subendocardial	23 (32.8%)		
Diffuse	5 (7.2%)		
LGE extent (g)	55.9 (37.3, 81.3)	10.2 (3.2, 25.2)	< 0.001
LGE extent (%)	49.4 (37.4, 63.8)	11.4 (4.5, 21.6)	< 0.001

CA: cardiac amyloidosis; HCM: hypertrophic cardiomyopathy; AL: light-chain amyloidosis; ESV: end-systolic volume; EDV: end-diastolic volume; LVEF: left ventricular ejection fraction; MWT: maximal wall thickness; LGE: late gadolinium enhancement

The patients with CA were older and had higher NYHA functional classes than the HCM patients. The HCM group had a higher body mass index. Moreover, more hypertensive patients were found in the HCM group than in the CA group. The NT-proBNP and troponin T levels were markedly higher in the CA group than in the HCM group (log NT-proBNP: 8.5 ± 1.1 vs. 7.0 ± 1.0; log troponin T: 4.6 ± 0.7 vs. 3.0 ± 0.9; *p* < 0.001 for both).

### Traditional CMR parameters

The ESV index was higher in the CA group than in the HCM group (CA vs HCM: 26.6 ± 9.5 vs 23.9 ± 9.2, *p* = 0.029), while the EDV index was higher in the HCM group (49.6 ± 11.4 vs 59.8 ± 13.8, *p* < 0.001). The LV mass index was comparable between the two groups (43.3 ± 13.3 vs 45.6 ± 17.5, *p* = 0.25), whereas the MWT was higher in the HCM group than in the CA group (15.4 [14.5, 17.6] vs 18.5 [16.2, 21.1], *p* < 0.001). CA patients displayed a remarkably higher LGE extent than HCM patients (55.9 [37.3, 81.3] g vs 10.2 [3.2, 25.2] g, *p* < 0.001).

### Multistep texture feature selection and dimension reduction

The multistep texture feature selection and dimension reduction process is described in Additional file 1: Fig. S1. In total, we extracted 837 texture features from 6 feature groups, including first-order features, gray level cooccurrence matrix (GLCM), gray level dependence matrix (GLDM), gray level run length matrix (GLRLM), gray level size zone matrix (GLSZM), and neighboring gray tone dependence matrix (NGTDM). Definitions and interpretations of the texture features are presented in the Additional file 1:. Based on the ICCs for intra- and interobserver reproducibility, 614 features with ICCs < 0.75 were first excluded. The Boruta algorithm, based on the random forest machine learning algorithm, further confirmed 51 important features. Then, a correlation matrix was calculated for these features to eliminate collinearity at the level of |rho|≥ 0.9, leading to a further reduction from 51 to 20 features. These 20 features were finally fed into the LASSO algorithm, resulting in the 7 most important and independent texture features used for model fitting.

### Selected texture features

The included set of features consisted of four wavelet-transformed features (GLRLM-Long Run Emphasis (LRE) LHL; GLRLM-Short Run Emphasis (SRE) LHL; GLRLM-LRE LLH; and GLDM-Small Dependence High Gray Level Emphasis (SDHGLE) HLH) and three original features (First order-Skewness; GLDM-Large Dependence Emphasis (LDE); GLCM-Correlation). All seven texture features showed significant differences between CA and HCM (Table 2). Correlation analyses suggested that GLRLM-SRE LHL was well correlated with GLRLM-LRE LHL (ρ = -0.85) and GLDM-LDE (ρ = -0.65). GLDM-SDHGLE HLH had moderate correlations with GLRLM-LRE LLH (ρ = -0.41), GLRLM-LRE LHL (ρ = -0.5) and GLRLM-SRE LHL (ρ = 0.51). The other features had weak or no correlations with each other (Additional file 1: Fig. S2).

**Table 2:** Differences of selected texture features between CA and HCM

Image type	Feature class	Features names	CA	HCM	*P* value
Wavelet-LHL	GLRLM	Long run emphasis	6.639 (5.796, 7.310)	7.360 (6.721, 8.266)	< 0.0001
Wavelet-LHL	GLRLM	Short run emphasis	0.535 (0.510, 0.567)	0.504 (0.478, 0.530)	< 0.0001
Wavelet-LLH	GLRLM	Long run emphasis	143.8 (104.5, 192.4)	193.9 (148.7, 252.2)	< 0.0001
Wavelet-HLH	GLDM	Small dependence high gray level emphasis	0.143 ± 0.019	0.128 ± 0.016	< 0.0001
Original	First order	Skewness	0.323 ± 0.436	0.107 ± 0.447	< 0.0001
Original	GLDM	Large dependence emphasis	48.3 (38.8, 56.2)	55.6 (50.2, 60.0)	< 0.0001
Original	GLDM	Correlation	0.911 (0.858, 0.951)	0.819 (0.712, 0.908)	< 0.0001

GLRLM: gray level run length matrix, GLDM: gray level dependence matrix, GLCM: gray level co-occurrence matrix; H: high wavelet filter; L: low wavelet filter

### Diagnostic performance of the selected texture features

**Fig. 2:**
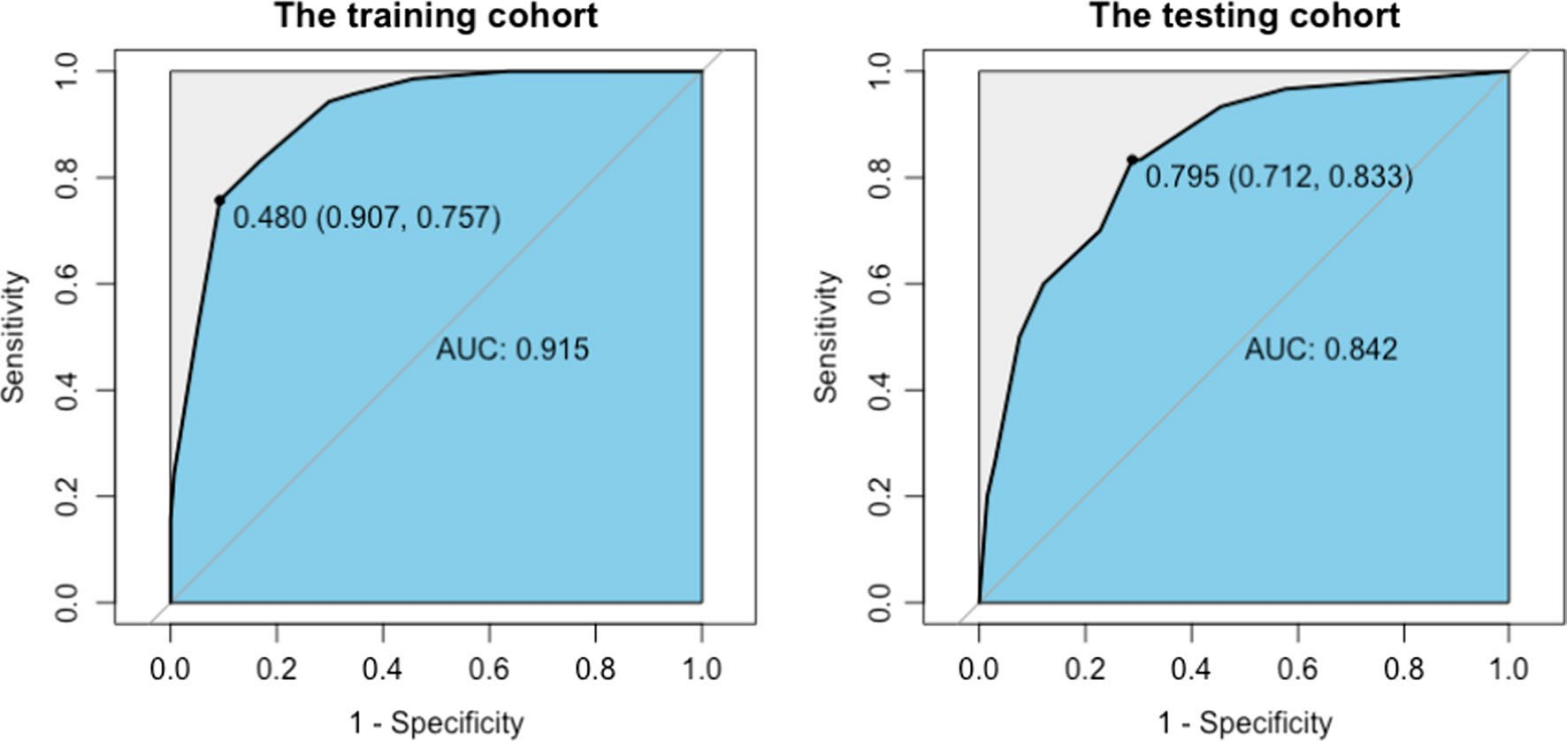
ROC curves for the radiomics model in the training and testing cohorts. ROC: Receiver operating characteristic curve; AUC: area under the ROC curve

The 7 selected texture features provided, in combination, a diagnostic accuracy of 86.0% (AUC = 0.915; 95% CI 0.879–0.951) in the training dataset and 79.2% (AUC = 0.842; 95% CI 0.759–0.924) in the testing dataset (Fig. 2). The differential diagnostic accuracy in the similar hypertrophy subgroup was 82.2% (AUC = 0.864, 95% CI 0.805–0.922) (Table 3). The significance of the difference between the AUCs of the TA model and LGE was verified by Delong’s test (TA model vs. LGE, *p* = 0.898).

**Table 3:** Diagnostic capacity of the selected tissue features for discriminating CA and HCM

	AUC (95%CI)	Accuracy	Sensitivity	Specificity	NPV	PPV
Training group	0.915 (0.879,0.951)	0.860	75.7	90.7	89.0	79.1
Testing group	0.842 (0.759,0.924)	0.792	60.0	87.9	82.9	69.2
Similar hypertrophy	0.864 (0.805,0.922)	0.822	83.5	81.0	83.1	81.5

AUC: the area under the curve, PPV: positive predictive value, NPV: negative predictive value

### Intraobserver and interobserver reproducibility

The ICCs of the 837 radiomics features ranged from 0 to 0.983. Among them, 614 features had an ICC < 0.75 and were excluded from further analyses. The reproducibility of the final selected 7 features was considered excellent (ICCs ranging from 0.757 to 0.949) (Table 4).

**Table 4:** Intraclass correlation coefficients for the intra- and interobserver reproducibility of the selected texture features

	GLRLM-LRE LHL	GLRLM-SRE LHL	GLRLM-LRE LLH	GLDM-SDHGLE LHL	First order-Skewness	GLDM-LDE	GLDM-Correlation
Intra-observer	0.949	0.893	0.855	0.847	0.880	0.794	0.773
Inter-observer	0.918	0.883	0.834	0.761	0.858	0.758	0.757

Abbreviations as in Table 2

## Discussion

Texture analysis is a postprocessing method to identify subtle tissue alterations and can be applied to standard and routinely acquired clinical CMR sequences. Our study demonstrated that TA was feasible and reproducible for detecting myocardial tissue alterations on T2-weighted images. The radiomics model constructed with texture features derived from this sequence had a great performance in differentiating between CA and HCM patients and was comparable to LGE quantification.

Our study indicated that the optimal combination of texture features had an accuracy of 86% for differentiating between CA and HCM. Considering HCM often shows extremely and heterogeneously increased wall thickness, we validated the diagnostic capacity of the TA model in patients with similar hypertrophy matched by LV mass index, age, sex, and MWT. This subgroup analysis showed that the TA model still had a great discriminative capacity with high sensitivity and specificity for CA and HCM.

LGE is the most popular and useful CMR sequence in clinical settings. However, the required administration of a gadolinium agent has limited some patients from undergoing this examination. For patients with systemic amyloidosis, the kidney is a common target organ other than the heart for amyloid infiltration. Thus, many CA patients may have impaired renal function due to amyloid deposition. These patients would be cautious about the use of gadolinium contrast agent. And the use of a gadolinium contrast agent is known to have the potential to cause renal sclerosis [25]. Thus, development of a non-contrast technique is very important to the diagnosis of CA.

There are many researchers seeking to develop novel and gadolinium-free techniques for the characterization of myocardial diseases. For example, Neisius et al. utilized texture features of the myocardium through native T1 mapping to discriminate between HCM and hypertensive heart disease [26]. Baessler et al. indicated that TA of non-contrast cine images allowed for the diagnosis of subacute and chronic ischemic scars with high accuracy [27].

TA can comprehensively and elaboratively analyze the spatial distributions of pixel gray levels in images, which further derives substantial quantitative texture features characterizing the underlying tissue texture [28]. These texture features, in combination with robust mathematical models, could represent reliable diagnostic tools [29] and overcome the limitations of subjective visual image interpretation. Moreover, this technique does not require the administration of contrast agent or the acquisition of additional sequences. It can be applied to standard and routinely acquired clinical CMR sequences. Further improvement of this technique may have the potential to eliminate the need for contrast agent administration and prompt TA to act as a useful ancillary diagnostic tool or prognostic marker.

Our study made an effort to apply TA to T2WI images. Because the T2WI is a routinely applied and widely accessible sequence for CMR in most institutions. A high signal intensity on T2WI is indicated to be correlated with myocardial edema and considered helpful for the diagnosis of myocarditis and acute myocardial infarction [30]. Hen et al. had previously reported that a high T2 signal was an independent predictor of life-threatening arrhythmic events in HCM patients [31]. Kotecha et al. demonstrated that myocardial edema was present in CA by histology and CMR T2 mapping. T2 signal was an independent predictor of death in light-chain amyloidosis (AL), suggesting that in addition to amyloidosis infiltration, myocardial edema possibly caused by amyloidosis fibril toxicity would be an additional mechanism contributing to mortality [32]. Our study demonstrated that texture features derived from T2WI could reflect tissue alterations of the myocardium in CA and HCM.

The most important texture feature for discriminating between CA and HCM on T2WI in our study was GLRLM-SRE LHL. A greater value of GLRLM-SRE represents finer textural textures, while a greater value of GLRLM-LRE indicates coarser structural textures. Both features are derived from the run-length feature matrix, which describes the number of gray level runs of various lengths. A gray level run is defined as a group of pixels that have the same gray-level value in a given direction. The length is the number of pixels within the run. The run-length matrix elements describe the number of runs of a specific gray level value, and a particular run length can be observed in the ROI [33]. Thus, the two features GLRLM-SRE and GLRLM-LRE can serve as a measure of tissue homogeneity. In our study, the CA group showed lower GLRLM-LRE and higher GLRLM-SRE values than the HCM group, suggesting that CA has a finer textural texture on T2WI. The possible reason for this might be correlated with the more pronounced myocardial edema present in CA [32]. The coarse texture of HCM might reflect tissue inhomogeneity, such as myocardial disarray and fibrosis. Thus, TA seems to be a useful quantitative tool for the identification of tissue alterations.

## Limitations

This study still had several limitations. First, since this was a retrospective study across several years, genetic testing was not available for the majority of the patients. And texture analysis of novel sequences, such as T1 mapping and T2 mapping, could not be performed. We plan to conduct prospective studies with these novel imaging techniques in the future. Second, the CA group patients was predominately (93%) the light-chain amyloidosis (AL). Even though there is little difference in signal features between the AL and the other subtype of amyloidosis, our study result is limited to the AL amyloidosis and needs to be confirmed in other subtypes of CA. Third, considering the histological alterations within a disease process are unlikely to be the same throughout the heart, the ROI used in this study can only reflect the regional characteristics of the myocardium. In addition, the sub-analysis of patients with similar hypertrophy was limited by the small sample size. Further well-designed prospective studies are necessary to determine the utility of these TA parameters for a more general application.

## Conclusion

In conclusion, our study demonstrated that texture analysis based on T2-weighted images can feasibly differentiate CA from HCM, even in patients with similar hypertrophy. The selected final texture features could achieve a comparable diagnostic capacity to the quantification of LGE. However, this is a preliminary study. Refined techniques and study design are needed to explore further in this area and confirm its clinical utility.

## Supplementary Information

**Additional file 1:** Additional information about the feature selection process.

## Data Availability

The datasets analyzed in the current study are available from the corresponding author on reasonable request.

## Abbreviations

CA: Cardiac amyloidosis
HCM: Hypertrophic cardiomyopathy
CMR: Cardiovascular magnetic resonance imaging
T2WI: T2-weighted imaging
TA: Texture analysis
LGE: Late gadolinium enhancement
EDV: LV end-diastolic volume
ESV: LV end-systolic volume
LVEF: Left ventricular ejection fraction
MWT: Maximal LV wall thickness
LASSO: The least absolute shrinkage and selection operator
GLCM: Gray level cooccurrence matrix
GLDM: Gray level dependence matrix
GLRLM: Gray level run length matrix
GLSZM: Gray level size zone matrix
NGTDM: Neighboring gray tone dependence matrix
LRE: Long run emphasis
SRE: Short run emphasis
SDHGLE: Small dependence high gray level emphasis
LDE: Large dependence emphasis
