# Supplementary material for: Texture analysis of T2-weighted cardiovascular magnetic resonance imaging to discriminate between cardiac amyloidosis and hypertrophic cardiomyopathy
Source: BMC Cardiovasc Disord. 2022 May 21;22:235. doi: 10.1186/s12872-022-02671-0 (PMC9124433; doi:10.1186/s12872-022-02671-0)
Supplement: Supplementary file 1 — Additional file 1: Additional information about the feature selection process. [file 12872_2022_2671_MOESM1_ESM.docx]

**Supplementary materials**

**R Packages Used for Statistical Analyses**

R packages used for statistical analyses were ggplot2 and corrplot for graphical visualization, and ROCR for receiver operating curve analyses. Boruta used for eliminating irrelevant features to stabilize the model; Glmnet for LASSO regression; Rpart and rpart plot fitting single classification trees; Caret delete the parameters with high correlation.

| **radiomte** | | | |
| --- | --- | --- | --- |
| **Feature class** | **Original** | **Wavelet-transformed** | **All** |
| **First order** | 18 | 144 | 162 |
| **Shape-related^*^** | 14 | - | 14 |
| **GLCM** | 24 | 192 | 216 |
| **GLDM** | 14 | 112 | 126 |
| **GLRLM** | 16 | 128 | 144 |
| **GLSZM** | 16 | 128 | 144 |
| **NGTDM** | 5 | 40 | 45 |
| **Total** | 107 | 744 | 828 851 |
| GLCM: gray level co-occurrence matrix; GLDM: gray level dependence matrix; GLRLM: gray level run length matrix; GLSZM: gray level size zone matrix; NGTDM: neighbouring gray tone dependence matrix.  * Shape-related statistics were removed, because the shape of the regions of interest was considered irrelevant for our analysis in this study. | | | |

**Table S1: Texture features extracted from each patient.**

**Figure S1 Feature selection and dimension reduction process.**

**
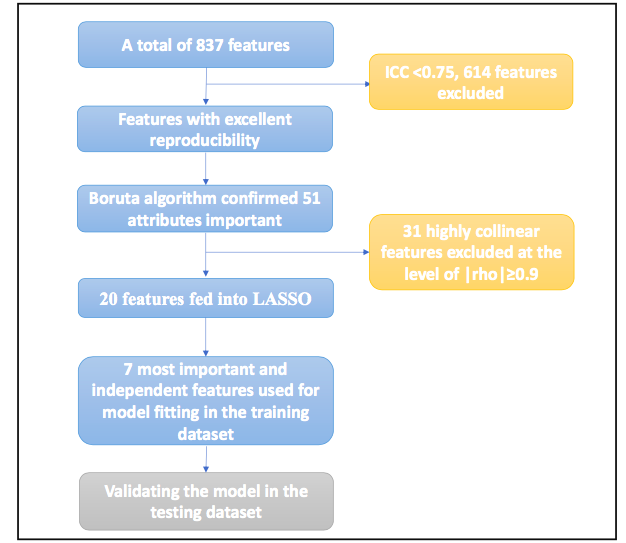
**

ICC: intraclass correlation coefficient, LASSO: the least absolute shrinkage and selection operator.

**Figure S2 Correlogram of the relationship among the selected texture features.**


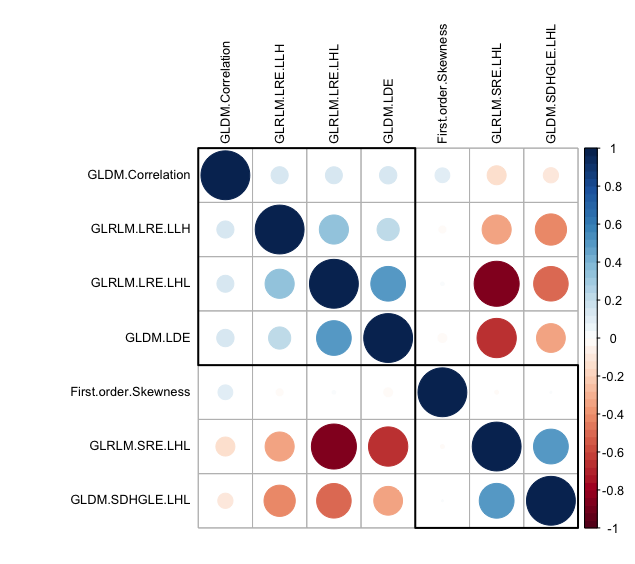


Smaller and/or lighter circles represent lower correlation. On the contrary, larger and/or darker circles indicate higher correlation. GLRLM: gray level run length matrix, GLDM: gray level dependence matrix, GLCM: gray level co-occurrence matrix; LRE: Long Run Emphasis; SRE: Short Run Emphasis; SDHGLE: Small Dependence High Gray Level Emphasis; LDE: Large Dependence Emphasis; H: high wavelet filter; L: low wavelet filter

**Figure S3: Classification Tree of the radiomic model**


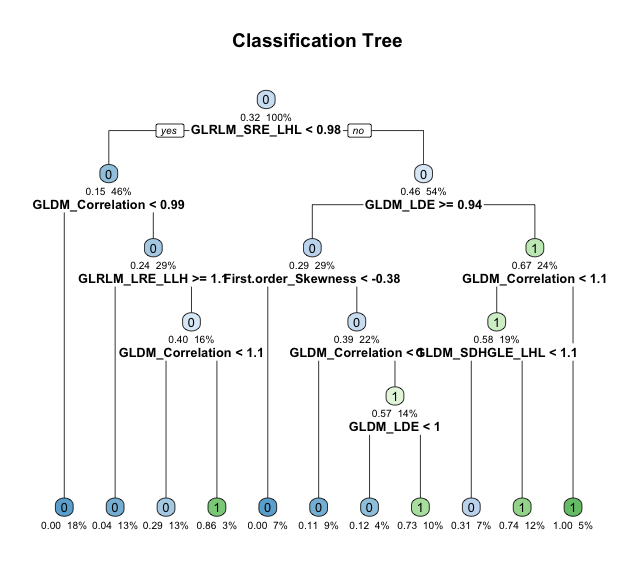


Abbreviations as in Figure S2.

**Figure S1: Wavelet transformation of the texture features.**


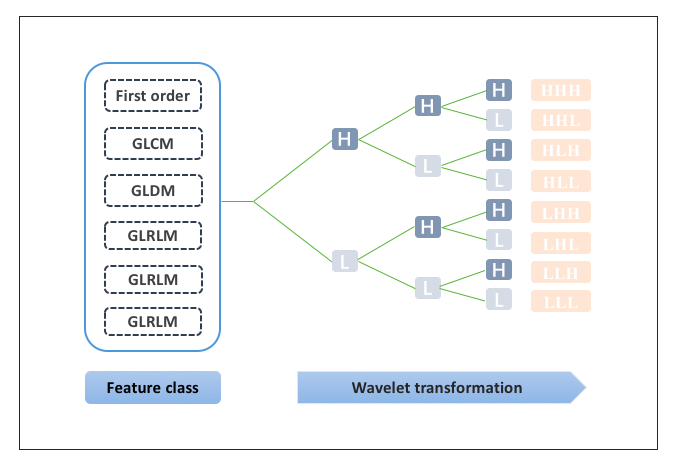


H: high wavelet filter; L: low wavelet filter, other abbreviations as in Table S1.

**Definition and interpretation of the selected texture features**

Definitions of the selected texture features were adapted from the pyradiomics library. Complete definitions and interpretations of all the features see <http://pyradiomics.readthedocs.io/en/latest/features.html>, last accessed September 28, 2020).

The pyradiomics community maintains the copyright for the definitions mentioned below (© Copyright 2016, pyradiomics community, [**http://github.com/radiomics/pyradiomics**](http://github.com/radiomics/pyradiomics) Revision eae15eff, last accessed September 28, 2020).

**1.Gray Level Run Length Matrix (GLRLM)**

A Gray Level Run Length Matrix (GLRLM) describes gray level runs, which are defined as the length in number of pixels, of consecutive pixels that have the same gray level value. In a gray level run length matrix ***P(i,j|θ)***, the ***(i,j)^th^*** element describes the number of runs with gray level ***i*** and length ***j*** occur in the image (ROI) along angle ***θ*** (Table S10).

Let:

***N_g_*** be the number of discreet intensity values in the image

***N_r_*** be the number of discreet run lengths in the image

***N_p_*** be the number of voxels in the image

***N_z_(θ)*** be the number of runs in the image along angle θ, which is equal to $\sum_{i=1}^{N_{g}} \sum_{j=1}^{N_{r}} \text{P}(i,j|\theta)$and 1≤N_z_(θ)≤N_p_

***P(i,j|θ)*** be the run length matrix for an arbitrary direction θ

***p(i,j|θ)*** be the normalized run length matrix, defined as $p(i,j|\theta)=\frac{\text{P}(i,j|\theta)}{N_{z}(\theta)}$

**ϵ** is an arbitrarily small positive number (≈2.2×10−16).

By default, the value of a feature is calculated on the GLRLM for each angle separately, after which the mean of these values is returned. If distance weighting is enabled, GLRLMs are weighted by the distance between neighbouring voxels and then summed and normalised. Features are then calculated on the resultant matrix. The distance between neighbouring voxels is calculated for each angle using the norm specified in ‘weightingNorm’

***a. Long Run Emphasis (LRE):***

$$\text{LRE}=\frac{\sum_{i=1}^{N_{g}} \sum_{j=1}^{N_{r}} \text{P}(i,j|\theta)j^{2}}{N_{z}(\theta)}$$

**LRE** is a measure of the distribution of long run lengths, with a greater value indicative of longer run lengths and more coarse structural textures.

***b. Short Run Emphasis (SRE):***

$$\text{ }$$

$$\text{SRE}=\frac{\sum_{i=1}^{N_{g}} \sum_{j=1}^{N_{r}} \frac{\text{P}(i,j|\theta)}{j^{2}}}{N_{z}(\theta)}$$

**SRE** is a measure of the distribution of short run lengths, with a greater value indicative of shorter run lengths and more fine textural textures.

**2.Gray Level Dependence Matrix (GLDM)**

A Gray Level Dependence Matrix (GLDM) quantifies gray level dependencies in an image. A gray level dependency is defined as the number of connected voxels within distance ***δ*** that are dependent on the center voxel. A neighbouring voxel with gray level ***j*** is considered dependent on center voxel with gray level ***i*** if ***|i−j|≤α***. In a gray level dependence matrix ***P(i,j)*** the ***(i,j)^th^*** element describes the number of times a voxel with gray level ***i*** with ***j*** dependent voxels in its neighbourhood appears in image (Table S12).

***N_g_*** be the number of discreet intensity values in the image

***N_d_*** be the number of discreet dependency sizes in the image

***N_z_*** be the number of dependency zones in the image, which is equal to $\sum_{i=1}^{N_{g}} \sum_{j=1}^{N_{d}} \text{P}(i,j)$

***P(i,j)*** be the dependence matrix

***p(i,j)*** be the normalized dependence matrix, defined as $p(i,j)=\frac{\text{P}(i,j)}{N_{z}}$

***a. Large Dependence Emphasis (LDE):***

$$\boldsymbol{LDE}=\frac{\sum_{i=1}^{N_{g}} \sum_{j=1}^{N_{d}} \text{P}(i,j)j^{2}}{N_{z}}$$

**LDE:** A measure of the distribution of large dependencies, with a greater value indicative of larger dependence and more homogeneous textures.

***b. Small Dependence High Gray Level Emphasis (SDHGLE):***

$$\boldsymbol{SDHGLE}=\frac{\sum_{i=1}^{N_{g}} \sum_{j=1}^{N_{d}} \frac{\text{P}(i,j)i^{2}}{j^{2}}}{N_{z}}$$

**SDHGLE**: Measures the joint distribution of small dependence with higher gray-level values.

**3.First order statistics**

These statistics describe the central tendency, variability, uniformity, asymmetry, skewness and magnitude of the attenuation values in a given region of interest (ROI), disregarding the spatial relationship of the individual voxels. As such, they describe quantitative and qualitative features of the whole ROI (PVAT). A total of 19 features were calculated for each one of the eight wavelet transformations and the original CT image (Table S6), as follows:

**Let:**

- ***X*** be a set of ***N_p_*** voxels included in the region of interest (ROI)
- ***P(i)*** be the first order histogram with ***N_g_***discrete intensity levels, where ***N_g_*** is the number of non-zero bins, equally spaced from 0 with a width.
- ***p(i)*** be the normalized first order histogram and equal to $\frac{\text{P}\boldsymbol{(i)}}{\boldsymbol{N}_{\boldsymbol{p}}}$
- **c** is a value that shifts the intensities to prevent negative values in X. This ensures that voxels with the lowest gray values contribute the least to Energy, instead of voxels with gray level intensity closest to 0. Since the HU range of AT (-190 to -30 HU) does not include zero, c was set at c=0. Therefore, higher energy corresponds to less radiodense AT, and therefore a higher lipophilic content.
- ***ϵ*** is an arbitrarily small positive number (≈2.2×10^−16^)

***a. Skewness:***

$$\text{Skewness}=\frac{\mu_{3}}{\sigma^{3}}=\frac{\frac{1}{N_{p}}\sum_{i=1}^{N_{p}} (\text{X}(i)-\overline{X})^{3}}{{(\sqrt{\frac{1}{N_{p}}\sum_{i=1}^{N_{p}} (\text{X}(i)-\overline{X})^{2}})}^{3}}$$

**Skewness** measures the *asymmetry* of the distribution of values about the Mean value. Depending on where the tail is elongated and the mass of the distribution is concentrated, this value can be positive or negative. (Where μ^3^ is the 3^rd^ central moment).

**4.Gray Level Co-occurrence Matrix (GLCM)**

In simple words, a GLCM describes the number of times a voxel of a given attenuation value ***i*** is located next to a voxel of ***j***. A GLCM of size ***N_g_***×***N_g_*** describes the second-order joint probability function of an image region constrained by the mask and is defined as P(***i,j***|***δ,θ***). The (***i,j***)^th^ element of this matrix represents the number of times the combination of levels ***i*** and ***j*** occur in two pixels in the image, that are separated by a distance of ***δ*** pixels along angle ***θ***. The distance ***δ*** from the center voxel is defined as the distance according to the infinity norm. For ***δ=1***, this results in 2 neighbors for each of 13 angles in 3D (26-connectivity) and for ***δ=2*** a 98-connectivity (49 unique angles). In order to get rotationally invariant results, statistics are calculated in all directions and then averaged, to ensure a symmetrical GLCM (Table S8).

Let:

**ϵ** be an arbitrarily small positive number **(≈2.2×10−16**)

**P(*i,j*)** be the co-occurence matrix for an arbitrary ***δ*** and ***θ***

**p(*i,j*)** be the normalized co-occurence matrix and equal to $\frac{\text{P}(i,j)}{\sum\text{P}(i,j)}$

Ng be the number of discrete intensity levels in the image

$\boldsymbol{p}_{\boldsymbol{x}}\boldsymbol{(i)=}\sum_{\boldsymbol{j=1}}^{\boldsymbol{N}_{\boldsymbol{g}}} \boldsymbol{P(i,j)}$ be the marginal row probabilities

$\boldsymbol{p}_{\boldsymbol{y}}\boldsymbol{(j)=}\sum_{\boldsymbol{i=1}}^{\boldsymbol{N}_{\boldsymbol{g}}} \boldsymbol{P(i,j)}$ be the marginal column probabilities

***μ_x_*** be the mean gray level intensity of ***p_x_*** and defined as $\boldsymbol{\mu}_{\boldsymbol{x}}\boldsymbol{=}\sum_{\boldsymbol{i=1}}^{\boldsymbol{N}_{\boldsymbol{g}}} \boldsymbol{p}_{\boldsymbol{x}}\boldsymbol{(i)i}$

***μ_y_*** be the mean gray level intensity of ***p_y_*** and defined as $\boldsymbol{\mu}_{\boldsymbol{y}}\boldsymbol{=}\sum_{\boldsymbol{j=1}}^{\boldsymbol{N}_{\boldsymbol{g}}} \boldsymbol{p}_{\boldsymbol{y}}\boldsymbol{(j)j}$

***σ_x_*** be the standard deviation of ***p_x_***

***σ_y_*** be the standard deviation of ***p_y_***

$$p_{x+y}(k)=\sum_{i=1}^{N_{g}} \sum_{j=1}^{N_{g}} p(i,j),\text{ where }i+j=k,\text{ and }k=2,3,\ldots,2N_{g}$$

$$p_{x-y}(k)=\sum_{i=1}^{N_{g}} \sum_{j=1}^{N_{g}} p(i,j),\text{ where }|i-j|=k,\text{ and }k=0,1,\ldots,N_{g}-1$$

$HX=-\sum_{i=1}^{N_{g}} p_{x}(i)\log_{2}(p_{x}(i)+\epsilon)$ be the entropy of ***p_x_***

$HY=-\sum_{j=1}^{N_{g}} p_{y}(j)\log_{2}(p_{y}(j)+\epsilon)$ be the entropy of ***p_y_***

$$HXY1=-\sum_{i=1}^{N_{g}} \sum_{j=1}^{N_{g}} p(i,j)\log_{2}(p_{x}(i)p_{y}(j)+\epsilon)$$

$$HXY2=-\sum_{i=1}^{N_{g}} \sum_{j=1}^{N_{g}} p_{x}(i)p_{y}(j)\log_{2}(p_{x}(i)p_{y}(j)+\epsilon)$$

For distance weighting, GLCM matrices are weighted by weighting factor W and then summed and normalised.. Weighting factor W is calculated for the distance between neighbouring voxels by $\boldsymbol{W=}\boldsymbol{e}^{\boldsymbol{-\|d}\boldsymbol{\|}^{\boldsymbol{2}}}$, where d is the distance for the associated angle.

***a. Correlation***

$$\text{Correlation}=\frac{\sum_{i=1}^{N_{g}} \sum_{j=1}^{N_{g}} p(i,j)ij-\mu_{x}\mu_{y}}{\sigma_{x}(i)\sigma_{y}(j)}$$

**Correlation** is a value between 0 (uncorrelated) and 1 (perfectly correlated) showing the linear dependency of gray level values to their respective voxels in the GLCM.
